# Using biomaterials to rewire the process of wound repair

**DOI:** 10.1039/c7bm00295e

**Published:** 2017-07-25

**Authors:** Anna Stejskalová, Benjamin D. Almquist

**Affiliations:** Department of Bioengineering, Royal School of Mines, Imperial College London, London SW7 2AZ, UK

## Abstract

Wound healing is one of the most complex processes that our bodies must perform. While our ability to repair wounds is often taken for granted, conditions such as diabetes, obesity, or simply old age can significantly impair this process. With the incidence of all three predicted to continue growing into the foreseeable future, there is an increasing push to develop strategies that facilitate healing. Biomaterials are an attractive approach for modulating all aspects of repair, and have the potential to steer the healing process towards regeneration. In this review, we will cover recent advances in developing biomaterials that actively modulate the process of wound healing, and will provide insight into how biomaterials can be used to simultaneously rewire multiple phases of the repair process.

## Introduction

The ability to heal wounds is intrinsically linked with our survival; wounds that fail to heal, such as diabetic foot ulcers, serve as a nurturing environment and entryway for infections. In turn, this leads to a lower 5 year survival rate than many common cancers including breast and prostate.^1^ Due to this critical link between wound healing and survival, mankind has aimed to develop strategies to promote healing since ancient times.^2^,^3^ In *circa* 2650 BC, a time when diseases were still thought to be of mystical origin, the Egyptian high priest and physician Imhotep developed an evidence-based guide for treating wounds that includes using bioactive substances such as honey and copper. Many of these details are recorded in written form on the Edwin Smith Papyrus, an ancient text that dates to *circa* 1600 BC.^3^ The impact of this effort to heal wounds helped lead to Imhotep’s eventual elevation to Deity of Medicine and Healing,^4^ an extremely rare honour and quite an illustrious example of the profound importance placed on wound healing.

If we fast forward over 4500 years, honey and metal ions are still key bioactive constituents in some of our most advanced wound dressings (*e.g.* MEDIHONEY, ACTICOAT).^5^ It should be noted that both of these strategies are aimed at treating infections in the wounds, and demonstrate the exceptional insights of the ancient Egyptians and the power of these natural substances. However, if we look to advanced bioactive strategies that are clinically approved for treating failures and deficiencies in the underlying biological process of wound repair, our toolbox is comparatively empty. Unlike diseases such as cancer, where methods for patient stratification, targeted therapies for specific mutations, and advanced combination therapies are common,^6^–^10^ the field of wound repair is still searching for answers in all of these areas.^11^

Part of the reason for this lack of targeted therapies is that outside of embryonic development, wound repair is one of the most complex biological processes our bodies must perform.^12^ Following injury, our tissues must simultaneously kill off infection, remove damaged and dying cells, and grow healthy replacement tissue.^13^–^15^ This process is tightly regulated, and involves a multitude of distinct cell types that coordinate with each other over time.^16^ Breakdown of this process at a multitude of different time points along this complex path can result in failure of the wound to heal. Furthermore, the natural outcome of healing in humans results in the formation of a scar; many times large area wounds such as burns lead to seriously debilitating complications from scarring including restricted joint mobility, loss of sensory ability, and trouble with temperature regulation.^17^ Taken together, the major impact of non-healing wounds and excessive scarring has given rise to substantial effort to develop advanced wound therapies that guide tissues towards successful repair and regeneration.

Over the past 15 years, biomaterials have rapidly become a key enabling technology in this push to develop advanced strategies for wound care.^11^ They provide methods for controlling the delivery of multiple therapeutics over time, provide supportive matrices for cellular growth, establish a barrier against infection, and bias the local microenvironment towards more regenerative outcomes. In this review, we will discuss recent advances in biomaterials design for actively modulating wound repair and regeneration, covering key aspects from initial injury through healing and demonstrating how these approaches address specific aspects of the biology of wound repair. Readers interested in comprehensive reviews of additional topics such as biomaterials for dressings (developmental and clinical) and cell-based dressings/therapies are referred to several excellent texts and reviews.^5^,^18^–^25^

## Rewiring the process of wound repair

**Fig. 1:**
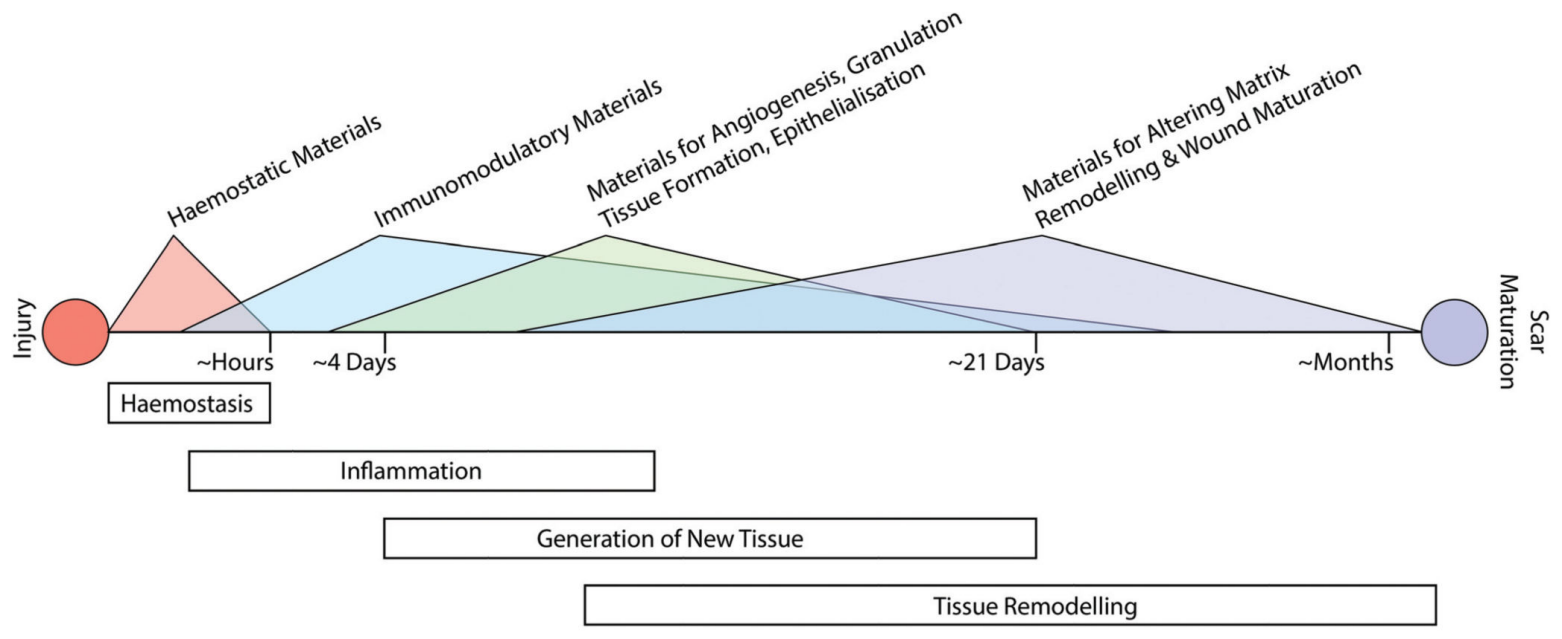
Following haemostasis, the process of wound healing follows a progression of events including inflammation, generation of new tissue, and remodelling of the nascent tissue; these phases occur over timescales that range from hours to many months. Biomaterials can be used to augment the natural repair process at all stages of wound repair, depending on the aspect of the repair process that is looking to be modulated. Ideally, biomaterials are designed/chosen according to the underlying biological process that is being targeted – there is no determined set of biomaterials that are ideal for one aspect of the wound healing process. This diversity and complexity in options for biomaterials mirrors the complexity of the biology underlying the process of wound repair.

The classical path of wound repair includes overlapping stages of inflammation, generation of new tissue, and subsequent remodelling of this nascent tissue.^13^ These stages take place over dramatically different timescales; initial clotting and coagulation can take place over minutes, whereas the process of tissue remodelling can extend from a period of several months to a year (Fig. 1).^26^ Depending on the nature of the intervention that is required, it may be necessary to target multiple aspects of the repair process at various time-points throughout healing.

### Augmenting haemostasis

The process of haemostasis is the initial step of the inflammation phase and begins immediately following wounding (Fig. 1). While this aspect of wound healing is not necessarily a key consideration for wound healing disorders such as chronic wounds, it plays a vital role in traumatic injury and can have significant impact on the subsequent process of repair. This is especially acute in areas such as battlefield injury, where a recent study attributed 90.9% of potentially survivable battlefield deaths to lethal hemorrhage.^27^ To address this issue, a variety of materials-based approaches have been developed to promote haemostasis.^21^

Upon wounding of vascularised tissues, a complex series of events give rise to a thrombus or clot.^28^ As part of the early stages of this process, von Willebrand Factor (vWF) in blood binds to exposed collagen that was previously shielded by endothelial cells. This causes elongation of vWF and presentation of several cryptic binding sites. These include binding sites for components of the coagulation cascade (*i.e.* factor VIII) and surface receptors on platelets (*i.e.* GP1b). In turn, a complex and dynamic interplay proceeds that involves proteins in the coagulation cascade, platelets, and components of vessel walls.^28^ As part of this process platelets are linked together in an early clot *via* fibrinogen bridges to form a mesh network. These platelets subsequently contract to densify the nascent network, stabilise the clot, and in turn stop the bleeding.^28^

**Fig. 2:**
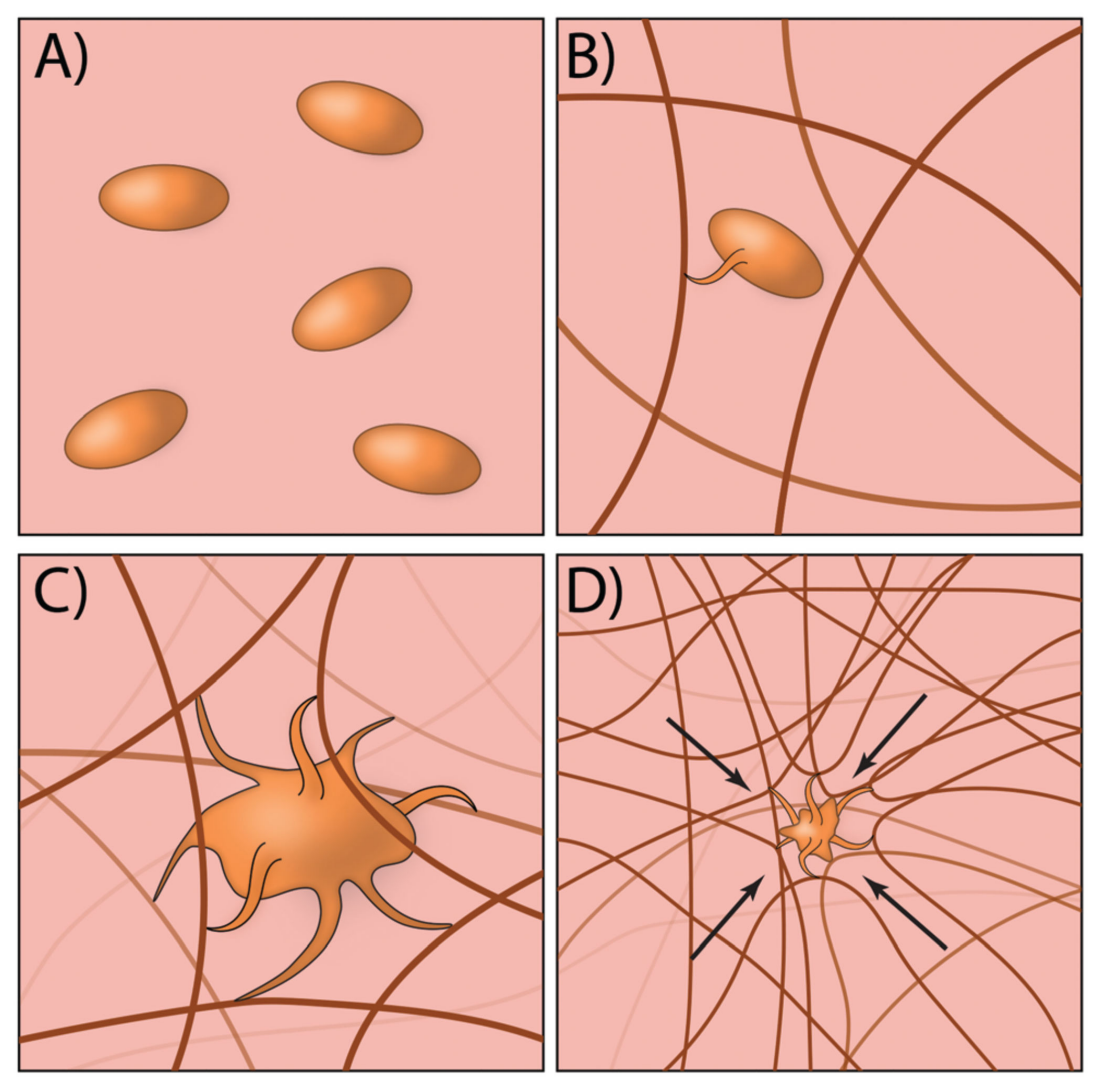
(A) PLPs circulate freely within blood due to no interactions with fibrinogen. (B) PLPs selectively bind fibrin protofibrils that begin to appear in the early stages of clot formation. (C) As the fibrin network grows, PLPs bond to multiple fibrin fibres. (D) The bonding interaction between PLPs and the fibrin network causes destabilisation and subsequent collapse of the network, in turn densifying the clot.

Traditionally, most exogenous approaches for promoting the formation of a clot simply act to form mesh networks or recruit clotting components to the site of need,^29^,^30^ but in large part are passive players in the process and cannot recapitulate key aspects of platelet function such as clot contraction. Recently, researchers have developed an innovative approach for creating platelet-like particles (PLPs) that have the ability to promote contraction (Fig. 2). The PLPs consist of ultra-low crosslinked poly(*N*-isopropylacrylamide-*co*-acrylic acid)microgels^31^ and were synthesised using a non-traditional precipitation polymerisation strategy. The microgels were subsequently decorated with antibody fragments that were evolved using phage display^32^ to recognise nascent fibrin fibres but not soluble fibrinogen. This is a key aspect of the design, since it enables PLPs to circulate freely within the vasculature but only become active when in the presence of an actively forming clot. Interestingly, the ability of PLPs to promote clot contraction arises without the need for active contraction machinery. The authors found that upon binding to nascent fibrin networks, the PLPs undergo significant deformation and bridging of multiple fibres within the network; computational modelling revealed that this bridging interaction leads to network destabilisation and subsequent collapse. In the end, this approach has an elegant simplicity that relies on rational materials design to passively recapitulate a key biological process that is normally actively driven.^33^,^34^

While a feature such as clot collapse is an important aspect of platelet function, platelets also play an integral role in initiating the process of wound repair. Upon activation and formation of a clot, platelets rapidly release (<10 minutes) the contents of their α-granules through an energy-dependent, SNARE-mediated process of exocytosis.^35^,^36^ The contents of these α-granules have been shown to consist of over 300 different proteins,^37^ creating a complex combination of signals that jumpstart the repair process. These proteins include numerous key cytokines including VEGF-A, PDGF-BB, FGF-2, IGF-1, HGF, CCL2, CXCL4, CXCL7, CXCL8 (IL-8), and CXCL12 (SDF-1α).^36^ Some of these, such as CXCL4 and CXCL7, are pre-dominantly expressed by platelets, whereas many of the other proteins released serve to supplement production by other cells in the local wound area.^36^

Despite this plethora of bioactive molecules that are released during the endogenous formation of a clot, many traditional biomaterials-based approaches do not recapitulate this bioactivity.^29^ While there are a variety of approaches to incorporate platelet lysate into biomaterials for promoting wound healing or tissue repair,^38^–^42^ these approaches generally do not address haemostasis and are more suitable for applications such as chronic wounds, stem cell differentiation, and angiogenesis. One approach that improves clotting times while also possessing some bioactivity is the use of keratin-based biomaterials.^43^ Researchers have demonstrated that kerateine biomaterials promote the attachment of platelets and increase fibrin polymerisation, in turn reducing clotting time.^44^–^46^ This behaviour is dependent on the presence of thiol groups, since keratose has been found to possess good blood compatibility.^47^ Interestingly, the process of keratin extraction leaves behind several residual growth factors that can be released into the local *in vivo* microenvironment.^48^ This feature likely contributes to the promotion of cell infiltration and the formation of granulation tissue when using kerateine materials,^44^ which is not observed with commercially available haemostats.

In addition to releasing cytokines that promote the growth of new tissue, platelets also release numerous cytokines that stimulate the immune system and shape the inflammatory response. This is done to ward off infection of the wound while also beginning the process of degrading and removing damaged and dying tissue. In order to promote haemostasis and address the risk of infection, researchers used the Layer-by-Layer (LbL) process to create self-assembled thin films that rapidly release thrombin within minutes to promote clotting, which is then followed by the antibiotic vancomycin over approximately 24 hours to help prevent infection.^49^ To delay the release of the small molecule antibiotic, the authors conjugated vancomycin to the hydrolytically degradable polyanion poly(β-l-malic acid). The authors demonstrated clotting and antibacterial activity *in vitro*, although it is still unknown how effective the combined function of the film is when used *in vivo*. However, in the past the authors have demonstrated that gelatin sponges coated with LbL films containing thrombin do indeed improve the rate of clotting in a porcine spleen laceration model.^50^ This focus on addressing both clotting and infection is especially important in large scale traumatic wounds, where bleeding needs to be rapidly stopped, wounds have a high likelihood of being dirty, and large regions of skin are compromised, which reduces the barrier to large scale infections and sepsis.^51^

### Shaping the inflammatory response

During this early stage of the inflammatory response, neutrophils are recruited and are important for minimising the risk of infection.^52^ However, the immune system as a whole plays a much larger role in coordinating and promoting successful tissue repair.^53^–^55^ While non-healing ulcers and hypertrophic scarring in humans display characteristic chronic inflammation, 56 macrophages play a key role in repair *via* scarring (both normal and pathological^57^) and wound regeneration across phylogeny;^58^–^62^ therefore it is the nature of the inflammation, not the inflammation itself, that is important for determining the outcome. For instance, macrophages are critical orchestrators of blastemas in both salamanders and zebrafish during limb and tailfin regeneration, respectively. 58,61 In both cases, ablating macrophages reduces proliferation in the mesenchymal tissue underlying the blastema. Fascinating work in the regenerating African spiny mouse *Acomys cahirinus* has also found evidence for the formation of a blastema during the regeneration of ear wounds, including the presence of macrophages in the local tissue.^63^ This suggests a conserved role of macrophages in orchestrating regeneration that likely extends through to the murid family of rodents.

Due to this important role of the immune system in modulating the repair response, there has been a growing interest in strategies to shape the nature of the inflammation phase. Researchers have been indirectly doing this by using mesenchymal stem cells (MSCs) for years; it has been demonstrated that MSCs that are injected into damaged tissue sites do not generally differentiate and remain long term, but instead alter the inflammatory response.^64^–^67^ Interestingly, MSCs have been shown to be of perivascular origin, co-expressing markers for pericytes.^68^ However, recent work using lineage tracing in mice has suggested that pericytes do not necessarily contribute to repairing damaged tissue *via* differentiation.^69^ Instead, the authors suggest that they may play a role during scar formation in cardiac and skeletal muscle. These findings fit well with the bodies of work on transplanted MSCs that suggest they play a key role in modulating the local microenvironment during repair, but not a significant role in long term engraftment. 70 Taken together, this widespread distribution of MSCs throughout vascularised tissues, but limited evidence of differentiation following damage, suggests a potential endogenous role in modulating the local tissue microenvironment following damage.

This strategy of local manipulation of the inflammatory response is rapidly becoming an attractive approach for modulating wound repair. However, instead of using MSCs to guide the response, researchers are rapidly developing new biomaterials-based approaches.^71^,^72^ In general, most approaches aim to bias the recruitment of macrophages towards anti-inflammatory “M2-like” macrophages over pro-inflammatory “M1-like” macrophages. M2-like macrophages are commonly characterised by Ly6C^low^CX3CR1^high^ expression, whereas M1-like macrophages display Ly6C^high^CX3CR1^low^ expression.^73^ However, it should be noted that there is a suggested method that is more detailed for classifying the diversity of macrophage subsets in experiments.^74^ In the past, the M2-like subset of macrophages has been shown to play an important role in promoting wound repair,^75^,^76^ while M1-like macrophages can prolong inflammation and negatively affect repair.^77^

One approach that has been explored to accomplish this selective recruitment of M2-like macrophages is the delivery of small molecule pro-resolving lipid mediators of inflammation named resolvins.^78^–^80^ Resolvins are synthesized from fatty acids (*e.g.* Omega 3) and promote a variety of pro-inflammation resolving processes, including the reduction of oxygen free radicals and phagocytosis of apoptosing leukocytes.^80^ One resolvin that has been used in several different biomaterials-based strategies is resolvin D1 (RvD1) and its epimer, aspirin-triggered resolvin D1 (AT-RvD1). In one set of studies, researchers demonstrated that treatment with RvD1 can reduce the pro-inflammatory response of chitosan scaffolds implanted into a subcutaneous air pouch.^81^ Follow-up work by the same group developed porous 3D chitosan scaffolds *via* temperature-mediated phase separation that released RvD1 to blunt the inflammatory response.^82^ In each case, the authors found a reduction in the release of several pro-inflammatory cytokines including IL-1α and IL-1β.

In other work, researchers have recently shown that AT-RvD1 released from poly(lactic-*co*-glycolic acid) (PLGA) scaffolds can bias the local immune cells towards a more pro-regenerative population.^83^ By releasing AT-RvD1 over the course of 7 days, the authors found a reduction in the number of inflammation-associated CD45^+^CD11b^+^Ly6C^+^Ly6G^+^ neutrophils at day 1, along with an increase at day 3 of a subset of neutrophils (CD49d^+^VEGFR1^hi^CXCR4^hi^) that assist with vascular remodelling. In addition, there was a general increase in the ratio of M2-like macrophages to M1-like macrophages. The levels of VEGF, IL-4, and SDF-1α, key pro-angiogenic and anti-inflammatory cytokines, were also increased at days 1 and 3. The end result of this strategy was an increase in the level of local vascular remodelling within the tissue.^83^

Another approach used to selectively recruit M2-like macrophages is the controlled release of cytokines that selectively target M2-like macrophages. In addition to expressing CX3CR1, M2-like macrophages express CXCR4 and S1PR3.^73^ In the past it has been shown that S1PR3 can promote transactivation of CXCR4, indicating a potential synergistic role in the recruitment of M2-like macrophages.^84^ To test this theory, researchers developed a dual affnity heparin-based hydrogel that controls the release of FTY720, a small molecule agonist of S1PR3, and SDF-1α, the ligand for CXCR4.^85^ The hydrogel consisted of a 9 : 1 ratio of PEG diacrylate and N-desulfated heparin methacrylamide, with bovine serum albumin (BSA) also incorporated during synthesis. BSA naturally sequesters small bioactive lipids and drugs, including FTY720, while SDF-1α binds heparin *via* a heparin-binding domain.^86^ Using this approach, the authors demonstrated a synergistic increase in the recruitment of M2-like macrophages when using the combination of FTY720/SDF-1α compared to either one by itself, along with an increase in vascular remodelling in the area surrounding the biomaterial.^85^

While none of the approaches discussed above have specifically demonstrated an improvement in wound healing, they present an attractive approach for exogenously shaping the inflammatory response. However, with that being said there is still much to understand regarding how to optimally manipulate the response. Both M1-like and M2-like macrophages have shown positive and negative impacts on wound healing, and their role and influence depend on when they are present during the process of repair;^87^ elucidating these complex interactions is therefore a pressing need. It will then be possible to combine these insights with an understanding of how macrophages respond to different wound matrices,^88^ in turn using synergistic insights to design biomaterials that steer wound repair towards regeneration.

It should be noted that in all the approaches discussed above, only the innate immune system is targeted. Recent work has also demonstrated an important role of the adaptive immune system in establishing a pro-regenerative microenvironment, specifically type 2 helper T cells (T_H_2 cells).^89^ In an elegant study exploring the impact of scaffolds synthesised from the cardiac tissue extracellular matrix on traumatic muscle injury, researchers found that in the presence of the biomaterial scaffolds, T_H_2 cells guide macrophage polarisation towards an M2-like, IL-4-dependent phenotype. In mice lacking T and B cells, this IL-4-dependent polarisation is lost and the biomaterial scaffolds give rise to a profile of macrophage polarisation that matches the saline control.^89^ This implication of a key role for the adaptive immune system in shaping pro-regenerative microenvironments is an exciting development, although more research is needed to understand how it can be further manipulated as part of a more comprehensive immunomodulatory approach to improve wound repair.

With that being said, no matter the strategy employed to shape the inflammatory response, the goal of this approach is to guide the growth of new tissue. In situations where wounds are not closed by surgical intervention, but instead heal *via* secondary intention, it is necessary to fill the wound site with vascularised granulation tissue.^90^ This new tissue then enables the migration of keratinocytes over the granulation tissue in order to close the wound.

### Promoting angiogenesis, the formation of granulation tissue, and epithelialisation

Granulation tissue is a highly vascularised tissue that begins growing into the wound site approximately 3–4 days following wounding.^91^ This tissue is composed of fibroblasts, macrophages, and neovasculature that move into the wound site in a coordinated fashion, and is coordinated by chemical as well as mechanical signals that are transmitted either by direct cell to cell contact or through the extracellular matrix (ECM). As discussed above, platelets release many of the signals necessary to orchestrate the onset of wound healing within 10 minutes of their activation.^35^ Studies have shown that while there is a delay of approximately 4 days between wounding and granulation tissue invasion, re-wounding followed by *de novo* fibrin matrix formation does not affect the rate of wound healing once the surrounding tissue has been activated.^92^ This suggests that the activation of the surrounding tissue by growth factors released from platelets is one of the rate-limiting steps in promoting the formation of granulation tissue.^92^

The diversity of cytokine signalling involved in driving the formation of granulation tissue is quite complex and covered in detail elsewhere.^93^,^94^ With that being said, there are several growth factors that are currently used clinically around the world to promote wound repair. One such growth factor is FGF-2, which is approved for use in Japan and China for treating non-healing dermal wounds and has a hand in promoting several aspects of wound repair. One source of FGF-2 is M2-like macrophages, which they release to promote angiogenesis and vessel maturation within the granulation tissue.^95^,^96^

While angiogenesis is a critical process for vascularising granulation tissue, FGF-2 has been shown to promote the vascularisation of new tissue independently of angiogenic sprouting; researchers found using two different neovascularisation models, a chick chorioallantoic membrane assay and a mouse cornea healing assay, that FGF-2-activated fibroblasts and myofibroblasts exert sufficient tension to rapidly expand existing vasculature *via* a mechanism that is independent of VEGF.^97^ Due to the applied tension, existing capillary networks become enlarged and blood vessel loops are pulled into the granulation tissue by activated fibroblasts. The neovessels contain a basal lamina and smooth muscle cells and are vWF-positive. Importantly from a materials perspective, this process can happen in collagen scaffolds when invaded by fibroblasts, but not in cross-linked scaffolds where contraction is prevented. It should be noted that after the first day, this mechanism is followed by VEGF becoming the dominant angiogenesis regulator.^97^ This switch between mechanical and chemical signalling is made more intriguing by recent work demonstrating that static 10% tensile strain induces cell cycle entry and sprouting of endothelial cells.^98^ Taken together, these findings present an intriguing strategy for developing materials with a similar effect to contracting fibroblasts. While we are not aware of any biomaterials-based strategies that specifically harness this mechanically driven vascularisation to promote wound healing, it is possible that it may occur during vacuum-assisted wound closure.^99^

**Fig. 3:**
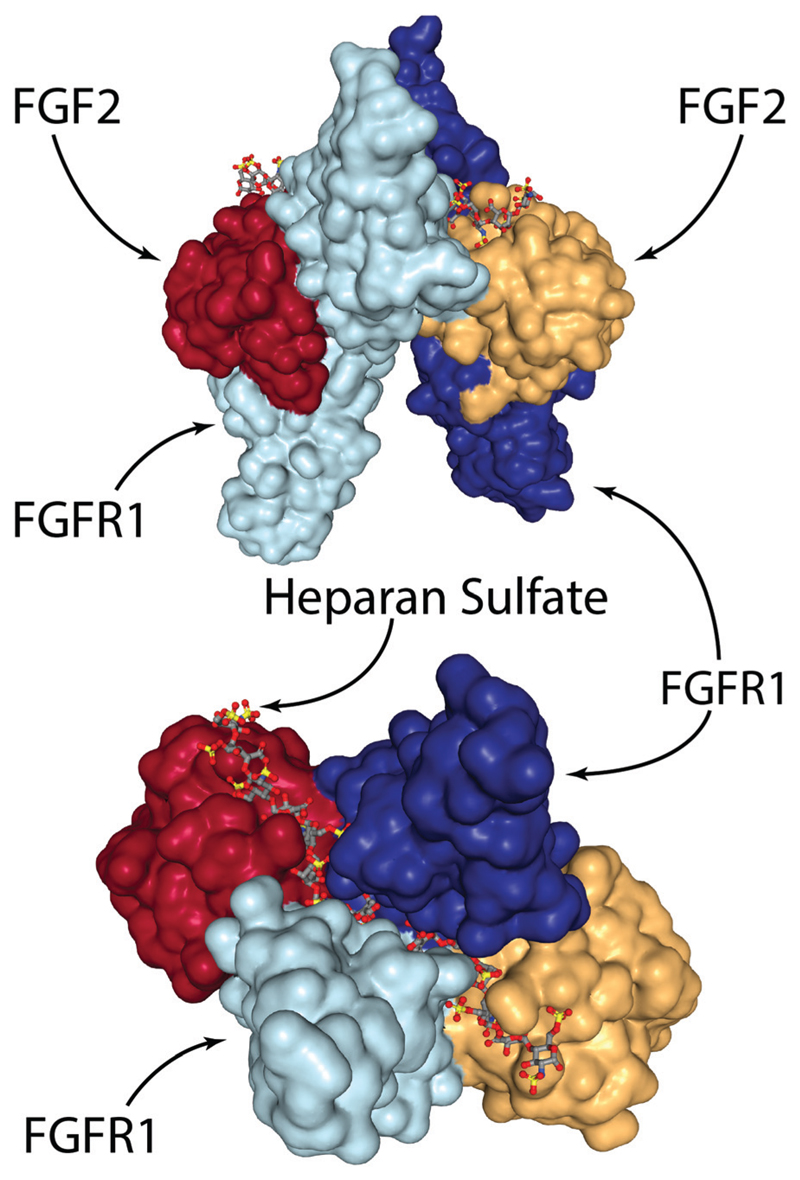
The FGF-2/FGFR1/heparan sulfate complex. Two FGF-2 molecules are tied together *via* a heparan sulfate chain that spans the middle of the FGFR1 dimer. Visualised from PDB 1FQ9^154^ using NGL viewer.

On the other hand, several groups have recently explored engineering-based methods for delivering FGF-2 to promote wound repair. When growth factors bind their cognizant receptor tyrosine kinases (RTKs), they generally do so as either preformed dimeric molecules (*e.g.* NGF, VEGF-A, PDGF-BB) or as pairs of growth factors in a larger growth factor-RTK complex (*e.g.* EGF, FGF-2).^100^ In the case of FGF-2, two RTKs are tied together *via* a heparan sulfate chain and two FGF-2 molecules (Fig. 3).^100^–^102^ In the complete FGF-2/FGFR1 complex, there is a well-defined spacing of the FGF-2 ligands. In one study, researchers created dimeric FGF-2 molecules linked together by a single PEG chain that matches the FGF-2 spacing in the complex.^103^ The authors found that a 2 kDa PEG spacer linking FGF-2 monomers resulted in the highest increase in signalling effcacy; 2 kDa PEG has a fully stretched length that is longer than 70 Å (the spacing in the FGF-2/FGFR complex between the cysteine residues on each FGF-2 molecule used for PEG linking), but a Gaussian chain length that is less than 70 Å. This leads to a spacing that is close to ideal, but requires slight steric stretching of the PEG chain to an extent that does not exceed available thermal energy. The authors showed that this engineered FGF-2 dimer leads to increased migration and proliferation of endothelial cells *in vitro*, along with increased formation of granulation tissue and density of blood vessels *in vivo* *via* a diabetic wound model in TallyHo/JngJ diabetic mice.^103^

**Fig. 4:**
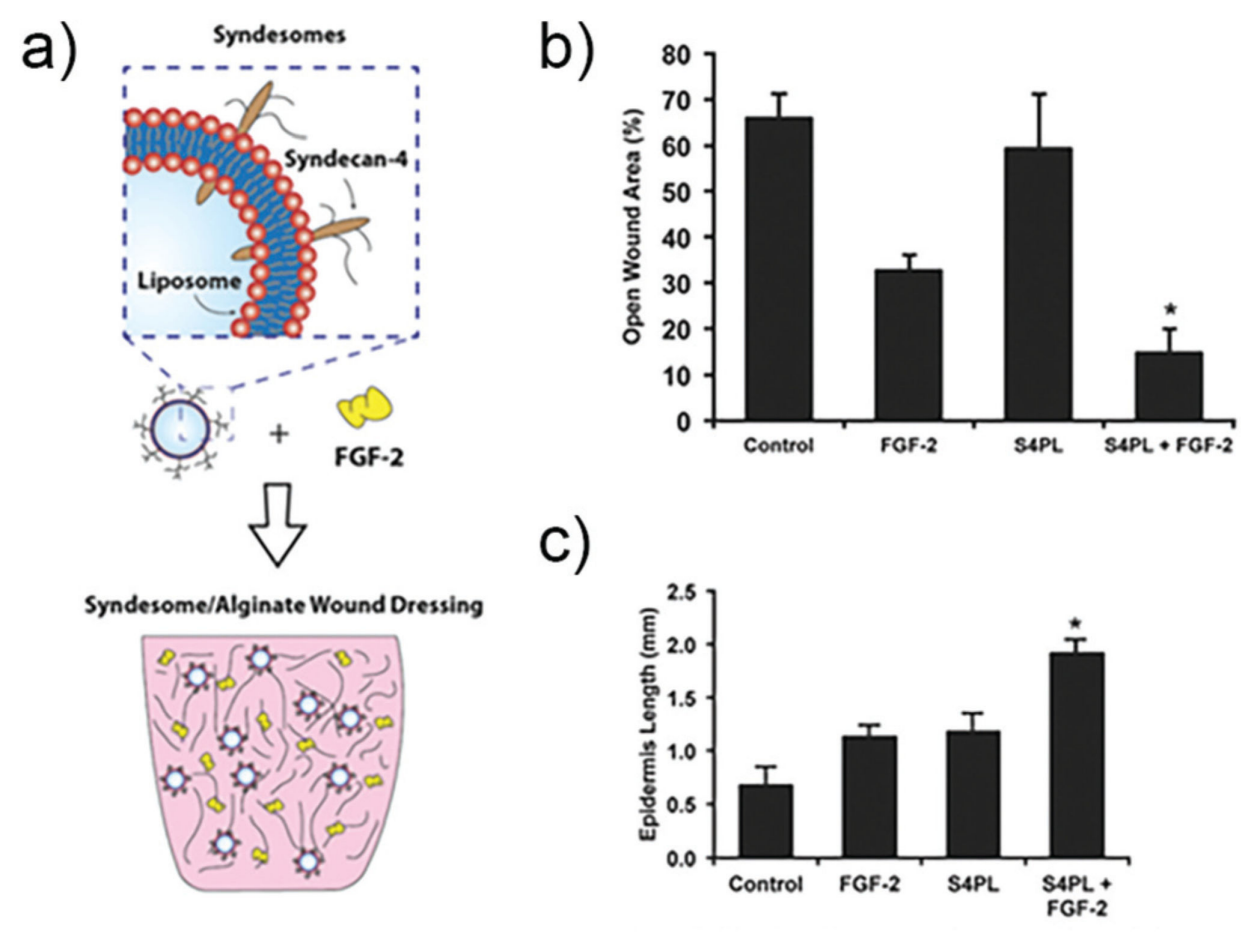
Structure of a syndesome and alginate wound dressings containing syndesomes and FGF-2. (b) Full thickness skin wound healing in ob/ob mice on high fat diet. Open wound areas after 14 days for untreated, FGF-2 treated, syndesome treated (S4PL), and syndesome + FGF-2 treated mice. (c) Quantification of epidermal growth beyond edge of wound at day 14. Adapted with permission from ref. 106. Copyright 2016 John Wiley and Sons.

In other work, researchers have explored the delivery of syndecan-4 with FGF-2 to enhance signalling (Fig. 4). Syndecan-4 is an important proteoglycan that is expressed on cell surfaces; the core protein of syndecan-4 is decorated with chains of heparan sulfate, which endows it with important functions related to cell signalling *via* regulation and concentration of growth factors, promoting the formation of focal adhesions, and directly facilitating signalling *via* its intracellular domain.^104^ In the case of FGF-2 signalling, syndecan-4 provides the heparan sulfate chain that integrates within the FGF-2/FGFR1 complex and helps facilitate signalling *via* the MAPK pathway. In the past, studies in knockout mice have shown that the loss of syndecan-4 results in delayed wound healing and a reduced density of vessels in the granulation tissue.^105^ Due to this importance in facilitating FGF-2 signalling, the authors of the study examined skin from diabetic patients and found that there is a reduction in the levels of syndecan-4 compared to non-diabetic skin.^106^ To address this deficiency the authors created liposomes that are decorated with syndecan-4, called ‘syndesomes’ (Fig. 4). When delivered in combination with FGF-2 from alginate dressings to wounds in diabetic ob/ob mice, this therapeutic approach resulted in faster wound closure and a higher density of blood vessels in the granulation tissue.

The preceding discussion begins to shed light on the importance of heparan sulfate proteoglycans in facilitating cell signalling and tissue repair. Unlike heparin, which is a highly sulfated form of heparan sulfate that is secreted predominantly by mast cells (an average of 2.3 sulfate groups per disaccharide for heparin, *versus* an average of 0.8 sulfate groups per disaccharide for heparan sulfate),^107^ heparan sulfates are ubiquitously expressed throughout our tissues as proteoglycans including syndecans, glypicans, and perlecan.^107^ Heparin is well known for its anti-coagulant activity, which is facilitated by a specific interaction with anti-thrombin that arises not through the high charge density of the sulfation pattern, but *via* a unique arrangement of sulfate groups and uronic acid epimers in a pentasaccharide group.^108^,^109^ On the other hand, heparan sulfates give rise to a highly diverse collection of proteoglycans with a variety of biological roles that depend on factors such as the sulfation pattern of the heparan sulfate chains.^107^

Recently, researchers exploited this sulfation-dependent behaviour to control the release of VEGF from biomaterial scaffolds to modulate wound repair. In this study, the authors selectively desulfated various moieties on heparin.^110^ They found that in addition to universally removing the key 3-*O*-sulfation necessary for binding to anti-thrombin, they were able to tune the binding and rate of release of VEGF. Removing either 6-*O*- or *N*-sulfation was found to significantly impact the affinity and rate of release of VEGF, whereas 2-*O*-sulfation had minimal impact. The authors then created completely desulfated heparin-PEG hydrogels *via* EDC crosslinking to control the delivery of VEGF. The completely desulfated heparin was synthesised by combining the desulfation protocols for *N*-, 6-*O*-, and 2-*O*-desulfation. By controlling the release of VEGF over 4 days, these gels were found to promote angiogenesis and the formation of granulation tissue in wounds using the diabetic db/db mouse model.^110^

While studies such as these have shown that VEGF and FGF-2 can promote angiogenesis and wound repair, many other growth factors have been explored as potential methods for promoting wound healing. In the USA, PDGF-BB cream has been approved by the FDA for treating neuropathic diabetic ulcers, but has been ‘black-boxed’ due to concerns about potentially promoting malignancy.^111^ In Cuba, an injectable version of EGF for chronic wounds has been developed and subsequently approved in various countries around the world.^112^ While these are demonstrations of the therapeutic potential of growth factors, they generally lack efficacy and therefore require large, supraphysiological doses.^113^ In order to reduce this need for high doses and increase efficacy, researchers have been developing methods for controlling the release of growth factors, while also enabling combinations of growth factors that are many times more efficient.^11^

**Fig. 5:**
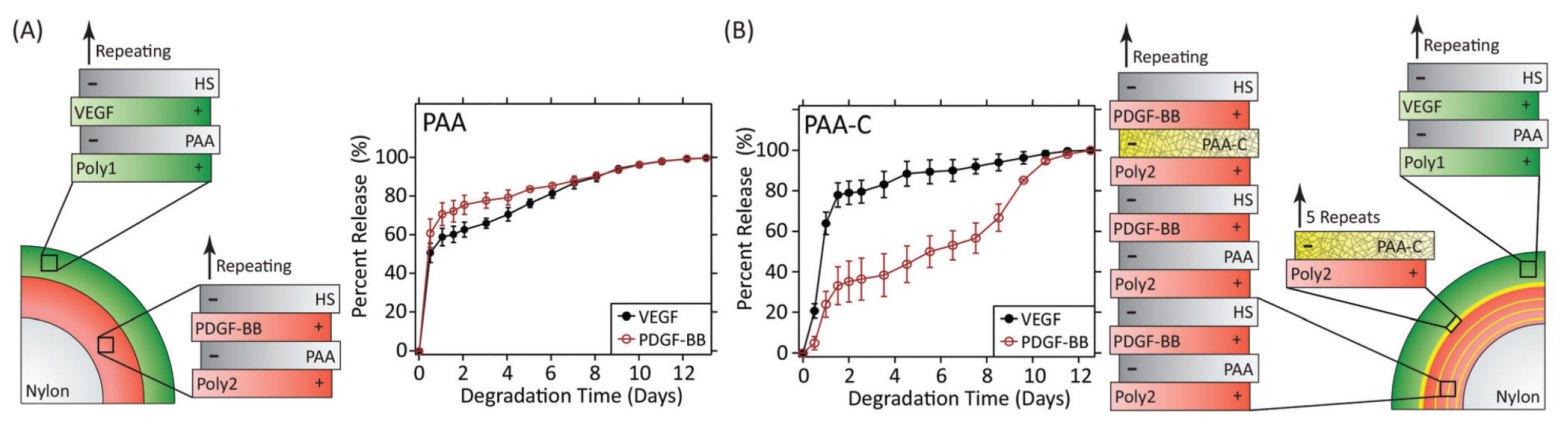
(A) LbL dressings assembled with VEGF-A165 and PDGF-BB lose distinct release profiles due to interdiffusion. (B) Layers of thiolated poly (acrylic acid) spontaneously form two-dimensional diffusion barriers within the film if spaced sufficiently far apart. The cross-linked layers serve as reversible diffusion barriers that enable individual control over the release kinetics of each growth factor. Adapted with permission from ref. 116. Copyright 2015 John Wiley and Sons.

A landmark study in 2001 demonstrated the power of combinations of growth factors that are released in coordinated fashion to promote, in this case, angiogenesis.^114^ In this study, VEGF-A was released quickly to promote angiogenic sprouting, while PDGF-BB was released slowly to promote the recruitment of mural cells to the newly formed vasculature in order to stabilise the network. Subsequent research has also shown that the kinetics of VEGF release alone impact the degree of angiogenesis.^115^ Recently, the LbL process has been used to create wound dressings that coordinate the release of VEGF-A165 and PDGF-BB to full thickness skin wounds in diabetic db/db mice.^116^ These dressings independently control the release kinetics of each growth factor *via* the formation of strategically placed two-dimensional diffusion barriers within the LbL films, which were formed *via* spontaneous disulfide formation within layers of thiolated poly(acrylic acid) (Fig. 5). When used *in vivo*, this strategy promoted an increase in vessel density and faster growth of the granulation tissue despite using over 300 times less growth factor than is used clinically.

While the VEGF and PDGF-BB combination has been shown to be effective at boosting angiogenesis and tissue growth, a recent study has expanded this combination to also explore the impact of the pro-angiogenic factor angiopoietin-2 (Ang-2), and the pro-maturation factor angiopoietin-1 (Ang-1).^117^ Using macroporous scaffolds consisting of alginate and PLGA, the authors found that releasing VEGF + Ang-2, followed by PDGF-BB or PDGF-BB + Ang-1 led to a higher degree of mature vasculature *in vivo*. In the end, this study begins to address the question of what combinations of growth factors are ideal for driving a biological response. In this case, the authors found a benefit from adding Ang-2, but did not see a significant difference after also adding Ang-1.^117^ Further exploring this area in the context of wound healing will begin to shed light on the relative importance of the various factors released by platelets during wound repair, and may enable the development of minimal combinations that promote robust and efficient wound repair. Given that in some cases platelet lysates and platelet-rich plasma may have clinical efficacy in promoting wound repair, and products based on this strategy are clinically approved (*e.g.* AutoloGel), a combinatorial, biomaterials-based approach is quite promising. However, it should be noted that a Cochrane Review from 2012 found no significant difference of autologous platelet rich plasma treatment in promoting the healing of chronic wounds over control groups,^118^ but noted that there is a lack of well-designed randomised controlled studies available. It is not unlikely, though, that the most efficacious method for promoting the healing of chronic wounds will require additional factors or temporal dynamics that are not possible *via* platelet-rich plasma alone, but can be incorporated into biomaterials-based approaches.^10^,^11^

The preceding examples demonstrate the impact of combining signalling from multiple growth factors to promote repair, but there are other cell signalling combinations that can also impact the process of repair. If we revisit the numerous roles of syndecan-4, it is possible to see the tangible link between RTK signalling and integrin signalling; syndecan-4 plays a critical role in both. It is well established that there is crosstalk and interaction between integrin and RTK signalling pathways,^119^,^120^ and this crosstalk has a role in directing numerous biological processes such as angiogenesis.^121^ Several groups have taken advantage of this interaction to promote synergistic signalling within wound sites to promote efficient healing.^122^ In one study, researchers engineered a recombinant peptide based on fibronectin that contained a factor XIIIa substrate fibrin-binding sequence, the 9th to 10th type III fibronectin repeat that contains the main integrin-binding domain, and the 12th to 14th type III fibronectin repeat that promiscuously binds numerous growth factors including VEGF-A165, PDGF-BB and BMP-2.^123^,^124^ The authors found a synergistic interaction between integrin and RTK signalling only when the domains were linked in close proximity. This synergistic interaction was then shown to promote the healing of numerous wounds, including critical size calvarial defects and full thickness skin wound in diabetic db/db mice.

**Fig. 6:**
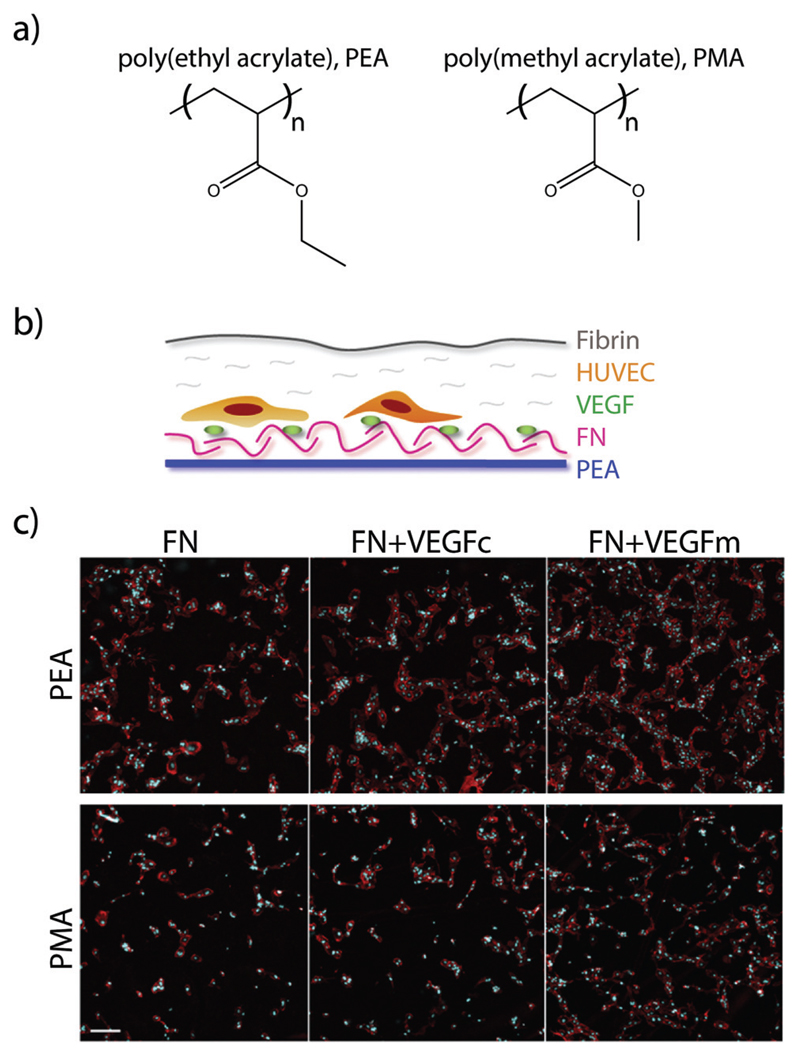
(a) Chemical structure of PEA and PMA. (b) HUVECs were seeded on surfaces coated with either PEA or PMA and fibronectin (FN), and then covered with a thin layer of fibrin. (c) Fluorescence images of HUVECs after 6 days culture on either FN surfaces, VEGF coated FN surfaces (VEGFc), or FN coated surfaces with VEGF in the media (VEGFm). Adapted from ref. 126 under permission of a creative commons license.

More recently, researchers have developed a materials-based approach for facilitating this synergistic interaction. The authors had previously demonstrated that surfaces of poly (ethyl acrylate) (PEA), but not poly(methyl acrylate) (PMA), give rise to fibronectin networks instead of globular fibronectin.^125^ In their recent study, they demonstrated that the process of network formation elongates and exposes the integrin and growth factor binding sites on fibronectin. This architecture of elongated fibronectin leads to close spacing between the integrin and VEGF binding sites, resulting in increased phosphorylation of ERK1/2, increased vascularisation and network formation *in vitro* (Fig. 6), and higher levels of endothelial cell recruitment into the poly(ethyl acrylate) scaffold *in vivo*.^126^ This approach for facilitating synergistic RTK-integrin signalling *via* a simple biomaterial is very attractive; the ability to coat the functional groups onto a variety of devices makes it applicable to a wide array of applications, while the use of natural proteins removes the cost and complexity associated with approaches based on protein engineering.

In general, approaches that target integrin signalling do it through providing a matrix with integrin binding sites that facilitate tissue ingrowth. While a vast majority of studies use the ubiquitous RGD sequence to facilitate integrin binding, it should be noted that different integrin binding sequences can be used to target specific integrins and drive different biological responses.^127^ For instance, past research has demonstrated that a set of fibronectin domains, FN III7-10, specifically targets α_5_β_1_ integrin.^128^ When compared to implant surfaces coated with RGD, this set of fibronectin domains enhanced osteoblastic differentiation of bone marrow stromal cells and promoted better osseointegration.^129^

In the context of wound healing, the physical nature and organisation of the biomaterial scaffold can have as much of an impact as the integrin binding sites. Recent research has developed an injectable microporous and biodegradable PEG gel that can accelerate the rate of wound healing.^130^ In this study, the authors created microparticles *via* water-in-oil immersions that were decorated with peptide substrates for transglutaminases (termed K and Q). When injected into full thickness skin wounds in mice in the presence of the transglutaminase factor XIII, the individual microspheres cross-linked into a microporous scaffold. In agreement with previous research demonstrating that microporous scaffolds that are formed *ex vivo* promote cell migration,^131^ the authors found that the *in situ* crosslinked microgels promoted faster wound healing than no treatment, uncrosslinked microgels, and solid scaffolds lacking the micropores.^130^ In the end, strategies such as these are intriguing for their use of natural enzymes from the coagulation cascade to give rise to biomaterials that promote faster wound healing. If combined with bioactive factors that can actively modulate the repair process, this approach may be very promising for simultaneously addressing multiple phases of wound repair.

### Downregulating detrimental overexpression in wound healing disorders

When it comes to wound healing disorders, such as diabetic foot ulcers, it is not necessarily enough to simply supply growth factors or artificial matrices to promote wound repair. In many cases, disorders such as these also have aberrant over-expression of proteins that are detrimental to the repair process. For instance, MMP-9 has been shown to be commonly upregulated in non-healing diabetic ulcers.^132^ To address this, researchers developed LbL dressings that facilitate controlled, localised delivery of siRNA to wound environments *in vivo*.^133^ These dressings were shown to significantly reduce the expression levels and MMP-9 activity in full thickness skin wounds of diabetic db/db mice (approximately 80% and 60% reduction at two weeks, respectively). In turn, this promoted significantly faster growth of granulation tissue and reepithelialisation.

In other work, researchers developed poly(thioketal urethane) (PTK-UR) scaffolds that release pH-sensitive, siRNA-loaded micellar nanoparticles locally within wounds upon triggered degradation of the scaffold *via* reactive oxygen species (ROS).^134^ The authors targeted prolyl hydroxylase domain protein 2 (PHD2), which has been shown to be upregulated in clinical diabetic ulcers; PHD2 is generally inactivated under hypoxic conditions, which prevents it from facilitating degradation of HIF-1α. In turn, this leads to expression of multiple pro-angiogenic cytokines including VEGF, Ang-1, and SDF-1. Using the db/db mouse model, it was shown that PHD2 knock-down gives rise to higher levels of HIF-1α, VEGF, density of vessels in the granulation tissue, Ki67+ proliferating cells, and rate of tissue infiltration.^134^

Another group of researchers also explored stabilising HIF-1α by reducing the production of iron-catalysed ROS and methylglyoxal. Previous studies have shown iron levels are increased in macrophages of patients with venous ulcers and that this leads to an increase in M1-type macrophages and ROS production.^135^ In other work, iron levels have been shown to be increased in the case of hyperglycemia and inhibit interactions between HIF-1α and its cofactor p300 *via* production of methylglyoxal.^136^ Deferoxamine (DFO) is an iron-chelating small molecule that has been approved by the FDA; in this study, the authors created an ethyl cellulose dressing that incorporated reverse micelles formed from non-ionic surfactants that contained the hydrophilic DFO and polyvinlypyrrolidone (used to prevent crystallisation of the DFO).^137^ This was necessary to facilitate transdermal delivery to diabetic pressure sores where the hydrophobic stratum corneum had not yet been compromised. Interestingly, the authors showed that in a pressure sore model in db/db mice, application of the DFO-eluting dressings prior to inducing wounding prevented the formation of pressure sores. This is a very interesting approach, as this dressing strategy can potentially be used as a preventative measure to reduce the formation of diabetic decubitus ulcers.

## A look towards the future

The examples given in the preceding section are but a fraction of the work within the past 5–10 years in the area of biomaterials for wound repair, which spans a wide range of topics from standard wound dressings through more advanced strategies for actively modulating repair.^21^,^22^,^24^,^138^,^139^ Inasmuch as the field has made significant advances towards actively modulating the process of wound healing, we are still presented with a lack of advanced biomaterials that comprehensively modulate the process of repair, along with technologies that have successfully made it through to the clinic. Part of the reason for this is that the underlying problems that give rise to defective wound healing are highly heterogeneous, and what will work for one subset of patients may likely be completely ineffective in another. In order to address this issue, new methods are needed to stratify patient populations and bias these advanced strategies for success.^140^ This will increase the likelihood of success at the clinical trial level, which has generally been rife with failures.^11^ New techniques, such as profiling macrophages^141^ or possibly non-invasive imaging strategies,^142^ may provide useful insights that will aid us in this task. However, we are still severely lacking in effective methods and biomarkers, especially when compared to diseases such as cancer.

These insights will also enable the biomaterials community to develop strategies that more comprehensively target key defects that act as bottlenecks to successful healing. To date, most strategies that have been developed target individual aspects or phases of the repair process. As biomaterials are developed that are more dynamic in nature, interacting in a bidirectional and synergistic manner with tissues,^10^ it will likely be possible to more adroitly steer the process of repair towards regeneration. These methods will surely need to target multiple aspects of the repair process including cell- and tissue-level signalling networks, along with the composition of the matrix. For instance, African spiny mice produce a provisional wound matrix that is enriched in fibronectin but low in collagen I and III. This low collagen content is facilitated by low expression levels along with high levels of proteases such as MMP-9.^63^ In contrast, mice that heal *via* scarring show high levels of collagen I and III during repair. This differential expression of ECM is recapitulated in many other contexts including modulating stem cell function and in cancer microenvironments.^143^–^147^

Understanding the fundamental biological impact of these differences, and to what extent they direct cellular behaviour *versus* being a result of it will provide key insights necessary to design new therapeutic biomaterials. However, hand-in-hand with these studies needs to be a push for more representative models of defective wound healing in order to rigorously test these new insights. Currently available *in vivo* wound models do not recapitulate the complex changes that occur in clinical wounds,^148^ making it difficult to predict how successful the translation to humans will be; as organ-on-a-chip systems increase in complexity, they may provide an interesting avenue for testing in human-relevant models.^149^,^150^ Because of this immense complexity, it is crucial that the biomaterials community works closely with biologists, bioinformaticians, and clinicians to drive new innovations and uncover new insights that are not possible in isolation. For instance, biomaterials-based approaches for modulating gene expression provide the possibility to transiently alter protein levels locally within a wound.^133^,^151^–^153^ This can be used to understand how specific biological changes affect key stages of wound repair, a task that is significantly more difficult or in many cases impossible using advanced techniques from biology including transgenic animals and strategies for gene editing (*e.g.* CRISPR/Cas9).

These new insights can then be incorporated into biomaterials-based treatments that are easily integrated into clinical settings. The ability to coordinate the release of multiple therapeutics over time from wound dressings and fillers reduces the barrier for successful patient compliance with challenging patient populations,^11^ is easily applied by nurses and physicians, and can be much more effective and tightly controlled than creams, sprays, and solutions such as platelet-rich plasma. In the end, we are confident that as the pace of innovation and development within the biomaterials continues to increase, we will see more and more advanced therapies make their way to the clinic, supplementing the current advanced wound care strategies that trace their roots back over 4500 years to Imhotep, the Egyptian God of Medicine and Healing.
